# Effect of thermoformed tray architecture on transfer accuracy of lingual brackets: A prospective clinical study

**DOI:** 10.1371/journal.pone.0341332

**Published:** 2026-01-27

**Authors:** Viet Anh Nguyen, Thi Quynh Trang Vuong, Thi Hong Thuy Pham, Thu Trang Pham, Viet Hoang

**Affiliations:** 1 Department of Orthodontics, Faculty of Dentistry, Phenikaa University, Hanoi, Viet Nam; 2 Private Practice, Viet Anh Orthodontic Clinic, Hanoi, Vietnam; 3 Department of Orthodontics and Pedodontics, Faculty of Dentistry, Hai Phong University of Medicine and Pharmacy, Hai Phong, Vietnam; 4 Department of Orthodontics, National Hospital Odonto-Stomatology, Hanoi, Vietnam; 5 Department of Orthodontics and Pedodontics, Faculty of Dentistry, Van Lang University, Ho Chi Minh City, Vietnam; University of Puthisastra, CAMBODIA

## Abstract

**Introduction:**

This prospective clinical study assessed whether thermoformed transfer tray architecture influences in-vivo positional fidelity of lingual brackets during indirect bonding, and whether the resulting deviations remain within clinically acceptable limits.

**Methods:**

A fully digital indirect bonding workflow was used. Bracket positions were planned on a virtual setup, and transfer trays were fabricated by thermoforming on 3D-printed models. Four tray designs were tested clinically under routine full-arch lingual bonding: a single-layer rigid tray (SR1) and three bilayer trays with a flexible inner liner of increasing thickness (BL1, BL2, BL3). After bonding, each bracket was digitized and superimposed onto the planned position using a local bracket-based coordinate system. Linear (mesiodistal, buccolingual, vertical) and angular (rotation, crown angulation, torque) deviations were calculated at the tooth level and compared across tray designs.

**Results:**

All trays achieved clinically acceptable transfer accuracy. All linear deviations remained below 0.5 mm at the group level, and rotation and angulation were generally within 2.0°. SR1 showed the most favorable buccolingual and rotational control but required the longest chairside delivery time. BL1 achieved acceptable accuracy with a shorter delivery time. Increasing liner thickness (BL2, BL3) did not improve precision and was associated with greater deviation in several dimensions.

**Conclusion:**

Tray architecture affected which axes were most vulnerable to error. Torque remained the least predictable dimension across all designs. Clinically, SR1 may be preferred in cases requiring strict control of rotation and buccolingual position, whereas BL1 offers a faster alternative for straightforward alignment without routine escalation to thicker bilayer trays.

## Introduction

Indirect bonding (IDB) has become a linchpin of contemporary digital orthodontics because it streamlines chairside workflows and improves the fidelity of bracket placement relative to freehand direct bonding. Across the labial literature, a consistent pattern emerges: linear transfer (mesiodistal, buccolingual, vertical) is typically more precise than angular transfer, with torque being the least accurate component [1,2]. Clinical trials and in-vivo evaluations of computer-aided jigs report sub-millimeter linear deviations but comparatively larger rotational errors, particularly in torque, and they repeatedly call for standardized coordinate systems and reporting thresholds to enhance comparability across studies [2,3]. Several head-to-head investigations also suggest that 3D-printed trays can deliver smaller linear errors than thermoformed guides on the labial side, although angular control remains the limiting dimension in both formats [4,5].

Evidence for lingual appliances, where bracket bases are smaller, undercuts are more pronounced, and enamel curvature complicates positioning, has expanded only recently. A prospective in-vivo study reported that double thermoformed trays fabricated on 3D-printed models achieved clinically acceptable transfer accuracy for lingual brackets, with workflow advantages that favor routine use [6]. Complementary comparative work indicates that thermoformed trays perform at least comparably to rigid and flexible 3D-printed guides while shortening chairside time, supporting their cost-effectiveness in everyday practice [7]. In parallel, a clinical case report demonstrated reliable multi-tooth transfer using a single rigid thermoformed tray for lingual bonding, reinforcing that simple thermoformed jigs can be sufficient when case selection and execution are controlled [8]. Together, these data suggest that thermoforming remains a strong contender alongside printed systems in lingual IDB.

Despite this progress, important knowledge gaps persist. Much of the comparative literature contrasts printed and thermoformed guides, while the architecture within thermoformed systems, for example, single-layer versus bilayer constructions and the influence of inner-liner thickness, remains relatively underexplored in controlled, head-to-head designs [3,9]. Complementing these gaps, recent within–3D-printed tray evaluations, contrasting fully vs partially enclosed lodgements, grouped vs single-tooth configurations, and one- vs three-piece segmentation, show design-dependent differences in transfer accuracy and chairside efficiency [10–12]. A further limitation across the literature is sample size. Many in-vivo studies enroll modest cohorts (tens of patients, a few hundred brackets), which constrains precision for tooth-group analyses and tail-risk estimation for torque-sensitive mechanics on the lingual surface [6,11–13].

Against this background, the present study focuses specifically on thermoformed tray designs for lingual IDB and their design-dependent performance. We evaluate transfer accuracy among trays that differ by construction (single-layer rigid versus bilayer soft-inner and rigid-outer) and by inner-liner thickness, using a standardized 3D superimposition workflow with tooth-surface alignment and bracket-based local coordinate systems. We test the null hypothesis that neither linear nor angular deviations differ across tray designs, and we additionally examine clinical acceptability against predefined limits and stratify outcomes by tooth group to reflect practical decision-making at the chair. By isolating the role of thermoformed architecture, this work aims to clarify when simple single-layer trays may suffice and when bilayer constructions with thicker soft liners confer measurable gains (or penalties) in positional fidelity, torque control, and overall reliability.

## Materials and methods

### Study design

This prospective observational cohort study protocol was reviewed and approved by the Institutional Review Board of Hanoi Medical University (approval code TT2301, dated 6 August 2023). All participants provided written informed consent prior to enrollment. Reporting followed STROBE guidance for observational studies. Adults seeking lingual orthodontic treatment were consecutively recruited from a private orthodontic practice (Cau Giay, Hanoi) between October 2023 and December 2024. Inclusion criteria were adults aged 18–50 years with complete permanent dentition and an indication for lingual orthodontic treatment, whereas exclusion criteria included a history of prior orthodontic treatment, active periodontal disease, dental malformations, or the presence of fixed prostheses in the dentition to be bonded. Because eligible lingual cases are relatively uncommon, convenience sampling was used, and all consecutive eligible patients during the recruitment period were invited. There was no randomization. For comparing four groups, an effect size f = 0.25 (medium), α = 0.05, and power (1–β) = 0.95 yielded a minimum of 70 brackets per group (total ≥280 brackets). This calculation was performed for one-way fixed-effects comparisons of mean deviations.

### Working model preparation and tray fabrication

**Fig 1:**
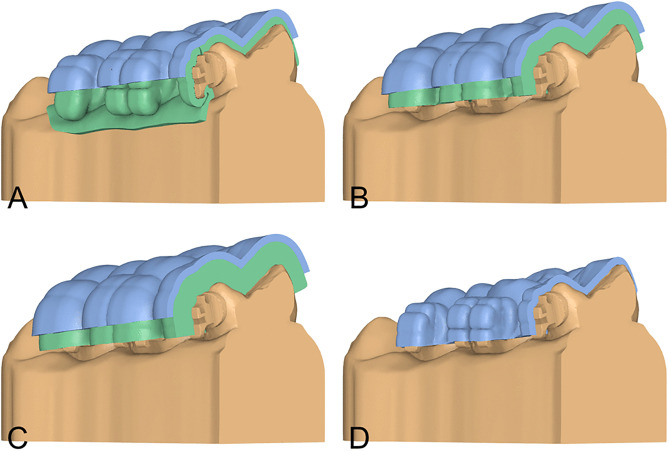
Thermoformed indirect bonding tray designs. The working model is displayed in yellow, the soft layer in green, and the rigid layer in blue. **(A)** BL1: bilayer tray with a 1.0-mm inner soft liner and a 1.0-mm outer rigid shell. **(B)** BL2: bilayer tray with a 2.0-mm inner soft liner and a 1.0-mm outer rigid shell. **(C)** BL3: bilayer tray with a 3.0-mm inner soft liner and a 1.0-mm outer rigid shell. **(D)** SR1: single-layer 1.0-mm rigid tray.

For each patient, a digital dental model was obtained with an intraoral scanner (Medit i700, Korea), and a virtual bracket setup for the lingual appliance was completed in dedicated software (Autolign, version 1.6; Diorco, Gyeonggi-do, Korea) according to the individualized treatment plan. Four thermoformed tray designs were fabricated and referenced thereafter as follows: a bilayer tray with a 1.0-mm inner soft liner + 1.0-mm outer rigid shell (BL1), a bilayer tray with a 2.0-mm inner soft liner + 1.0-mm outer rigid shell (BL2), a bilayer tray with a 3.0-mm inner soft liner + 1.0-mm outer rigid shell (BL3), and a single-layer 1.0-mm rigid tray (SR1) (Fig 1A-1D). The inner soft liners were Bioplast sheets, and the rigid layers were Biocryl sheets (Scheu-Dental, Iserlohn, Germany).

Working models carrying the virtually positioned brackets were prepared with differential management of bracket-wing undercuts to reflect tray behavior. In the BL1 group, bracket wings were left unblocked so that the soft liner could flow and grip around the bracket for secure seating. In contrast, for BL2, BL3, and SR1, bracket-wing undercuts were virtually blocked to avoid excessive mechanical interlock that might impede tray removal with thicker or rigid constructions. The models with brackets were then printed on a digital light processing 3D printer (Photon D2, Anycubic, Shenzhen, China) using Dental Model resin (Anycubic) and post-processed per the manufacturer’s instructions. Trays were formed on the printed models with a pressure-molding unit (AX-PMU4; IRIS, Tianjin, China) following each material’s recommended heating and forming protocol. For BL1, trimming preserved the full wrap of the soft liner around the bracket, including the undercut below the gingival wings, to maintain the intended retention; for BL2, BL3, and SR1, gingival margins were trimmed to expose the gingival wings of the brackets, reducing interlock and facilitating clean tray removal. This design-specific undercut blocking and trimming protocol was chosen to ensure secure seating while keeping tray removal clinically feasible; however, differences in undercut engagement may also influence tray retention and thus removal time across designs.

### Clinical bonding procedure

Lingual brackets (ADB; Medico, Gyeonggi-do, Korea) were inserted into the trays prior to clinical use. Teeth with severe crowding where brackets could not be seated, or brackets that became loose within the tray during insertion, were excluded from the analysis and removed from the working model. Each tray was sectioned into three segments, consisting of one anterior and two posterior parts, to facilitate seating and removal.

Before bonding, lingual enamel surfaces were cleaned with fluoride-free pumice, rinsed, and dried. The enamel was etched for 15 seconds using a 37% phosphoric acid gel (FineEtch; Spident, Korea), followed by application of a universal primer (Assure Plus; Reliance, Illinois, USA). Brackets were loaded with a light-cure adhesive (GoTo; Reliance, Illinois, USA), and trays were seated on the teeth under firm occlusal pressure. Each bracket was light-cured for 40 seconds using an LED curing unit (LedF; Woodpecker, Guilin, China).

Tray removal was performed according to the tray type. For bilayer trays, the outer rigid shell was removed first, followed by careful detachment of the inner soft liner. For the single-layer rigid tray, partial trimming of the tray around each bracket was carried out using a fine diamond bur (CD-53F; Mani, Utsunomiya, Japan) in a high-speed handpiece to facilitate subsequent removal. After all trays were removed, excess adhesive was finished and polished with silicone polishers (OneGloss; Shofu, Tokyo, Japan). A post-bonding intraoral scan was then acquired using the i700 scanner (Medit) for transfer accuracy evaluation. Bonding time was recorded for each patient from the placement of the first tray until removal of the last tray, and the number of bracket failures was documented simultaneously.

### Three-dimensional registration and deviation analysis

The initial virtual bracket setup served as the reference, and the post-bonding intraoral scan served as the target. The maxillary and mandibular arches were first aligned to allow segmentation of individual teeth, after which all measurements were performed with a local best-fit superimposition on a tooth-by-tooth basis to minimize errors from scan distortion when multiple teeth are matched simultaneously, especially because the presence of brackets on the digital scans can introduce surface artifacts and bending distortions [14,15]. Only tooth crowns and brackets were retained for analysis, and all soft tissues were removed. Brackets with defective scans, such as indistinct slot edges or missing parts of the bracket body, as well as brackets that failed during bonding, were excluded from the analysis.

**Fig 2:**
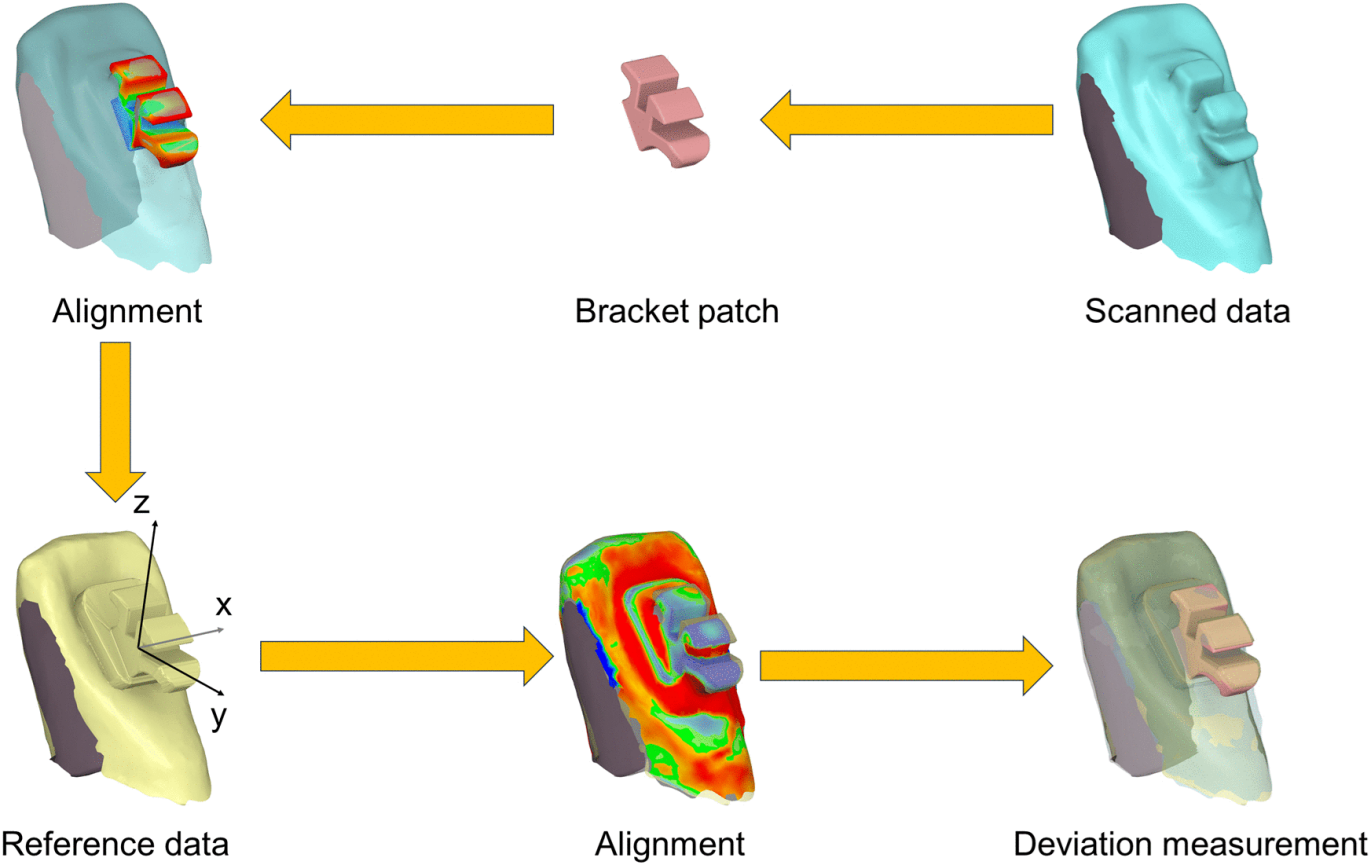
Workflow for deviation measurement. The design file of the bracket body was aligned to the post-bonding scan. Each tooth was then aligned to its reference using only tooth-surface data, with a local bracket-based coordinate system defined at the bracket body. Deviations were obtained by expressing the target bracket in the reference tooth’s local coordinate system.

For each tooth, a local bracket-based coordinate system was defined with the origin at the bracket body. The x-axis followed the mesiodistal direction along the bracket slot edge, the y-axis followed the buccolingual direction, and the z-axis followed the vertical occlusogingival direction. The manufacturer’s design file of the bracket body (excluding the mesh base) was first aligned to the target in order to place the physical bracket accurately on the post-bonding scan. The target tooth was then aligned to the reference tooth using only tooth-surface data and not the bracket geometry. Bracket transfer deviation was obtained by expressing the target bracket in the reference tooth’s local bracket-based coordinate system, and taking the resulting translational and rotational offsets as the deviation (Fig 2) [16].

Deviations were decomposed into six components. Translations were reported in the mesiodistal, buccolingual, and vertical directions. Rotations were reported as rotation around the vertical axis, angulation (tip) around the mesiodistal axis, and torque around the buccolingual axis.

### Statistical analysis

Normality was assessed with the Shapiro–Wilk test for each outcome; because distributions were non-normal, nonparametric methods were used throughout. Inter-rater reliability was evaluated on a prespecified subsample comprising 100 randomly selected brackets (6% of the total sample) using the intraclass correlation coefficient (ICC [2,1], two-way random-effects, absolute-agreement, and single-measurement) for each translational and rotational component, Bland–Altman analysis of mean signed differences with 95% limits of agreement, and Dahlberg’s method error. Baseline characteristics were compared as follows: categorical variables (sex, extraction indication, Angle classification) with chi-square tests; continuous variables (age, amount of crowding) with Kruskal–Wallis tests. Clinical handling outcomes were analyzed with Kruskal–Wallis for bonding time and chi-square for bond-failure rate.

Bracket-level deviation outcomes were analyzed overall and within tooth groups (anterior, premolar, molar). Only absolute values of the translational and rotational deviations were summarized to avoid the confounding effects of positive and negative signs. For clinical acceptability, each cell’s absolute deviation was tested against prespecified limits of 0.5 mm (linear) and 2.0° (angular) using one-sided, one-sample Wilcoxon signed-rank tests (H_1_: median < limit; H_0_: median ≥ limit). Comparisons among the four tray groups used Kruskal–Wallis tests; when significant, pairwise Mann–Whitney U tests were performed with Benjamini–Hochberg adjustment of p-values across pairs within each outcome. The overall significance level was α = 0.05 (two-sided). No additional multiplicity adjustment was applied across the six deviation components because these axes were predefined, mechanistically distinct measures of transfer accuracy. Analyses were conducted in Python (version 3.4; Python Software Foundation, Wilmington, DE, USA).

## Results

A total of 51 patients were included (n = 17 per group), contributing 1,709 bonded teeth and 1,682 analyzed brackets across the four tray groups (Table 1). There were no between-group differences in age (p = 0.163), sex distribution (p = 0.301), extraction status (p = 0.773), or Angle classification (p = 0.999). Crowding was comparable across groups (p = 0.279). Bonding time differed significantly among groups (p < 0.001), with SR1 having the longest bonding time (43 [42–52] min), followed by BL3 (38 [34–42] min), BL2 (35 [33–40] min), and BL1 (33 [30–36] min). Pairwise comparisons showed SR1 was significantly longer than all other groups, BL3 was significantly longer than BL1, and BL2 did not differ significantly from BL1 or BL3. Bond failure did not differ (BL1 0.7%, BL2 0.9%, BL3 1.6%, SR1 2.1%; p = 0.246).

**Table 1:** Baseline characteristics and clinical variables by tray group.

Variable	BL1 (n = 17)	BL2 (n = 17)	BL3 (n = 17)	SR1 (n = 17)	P value
Gender (F/M)	17/0	15/2	16/1	14/3	0.301
Extraction (Y/N)	8/9	7/10	8/9	10/7	0.773
Angle class (I/II/III)	14/2/1	14/2/1	13/3/1	14/2/1	0.999
Age (years)	27 [25 − 29]	25 [22 − 27]	29 [25 − 32]	30 [26 − 31]	0.163
Crowding (mm)	5.7 [2.2 − 8.4]	6.3 [3.5 − 11.7]	8.6 [4.8 − 9.6]	7.5 [6.0 − 11.6]	0.279
Bonding time (min)	33 [30 − 36]^a^	35 [33 − 40]^a,b^	38 [34 − 42]^b^	43.0 [42 − 52]^c^	< 0.001
Bond failure (n, %)	3 (0.7%)	4 (0.9%)	7 (1.6%)	9 (2.1%)	0.246
Bonded teeth (n)	429	431	426	423	
Analyzed brackets (n)	425	427	418	412	

Categorical variables (gender, extraction, angle class, bond failure) were compared using chi-square tests; continuous variables (age, crowding, bonding time) using Kruskal–Wallis tests. Continuous data are shown as median [IQR]. Two-sided α = 0.05. Superscripts indicate pairwise differences with Benjamini–Hochberg adjustment when the overall test is significant. BL1, bilayer tray (1.0-mm soft + 1.0-mm rigid); BL2, 2.0-mm soft + 1.0-mm rigid; BL3, 3.0-mm soft + 1.0-mm rigid; SR1 = single-layer 1.0-mm rigid tray.

**Fig 3:**
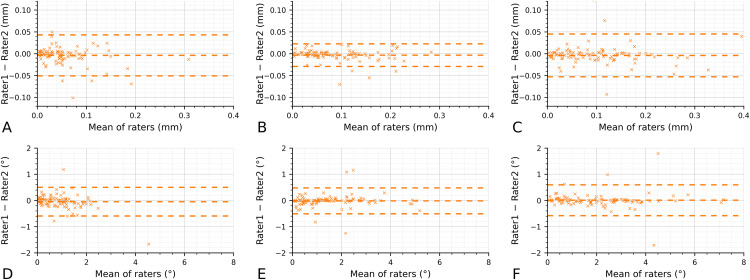
Bland–Altman plots for inter-rater agreement across six dimensions. A, Mesiodistal (mm). B, Buccolingual (mm). C, Vertical (mm). D, Rotation (°). E, Angulation (°). F, Torque (°).

Inter-rater agreement was high across all dimensions (Table 2). ICC ranged from 0.882 to 0.981. Systematic error was negligible for all outcomes, with bias near zero for translations (−0.004 to −0.003 mm) and angular measures (−0.046° to 0.010°). The 95% limits of agreement were narrow–approximately ±0.05 mm for translations and ±0.6° for angular measures–indicating close absolute agreement between raters (Fig 3A-3F). Dahlberg’s method error was small, on the order of 0.010–0.018 mm for translations and 0.178–0.211° for angular measurements.

**Table 2:** Inter-rater reliability for translational (mm) and angular (°) components (n = 100).

Dimension	ICC	ICC 95% CI	Bias	Limit of agreement	Method error
Mesiodistal (mm)	0.882	0.75–0.949	−0.004	−0.05–0.043	0.017
Buccolingual (mm)	0.976	0.954–0.99	−0.003	−0.029–0.023	0.010
Vertical (mm)	0.947	0.893–0.98	−0.004	−0.053–0.045	0.018
Rotation (°)	0.924	0.882–0.961	−0.046	−0.592–0.5	0.199
Angulation (°)	0.979	0.956–0.993	−0.015	−0.51–0.48	0.178
Torque (°)	0.981	0.959–0.996	0.01	−0.578–0.597	0.211

ICC, intraclass correlation coefficient; CI, confidence interval; bias, mean (rater1 − rater2).

**Fig 4:**
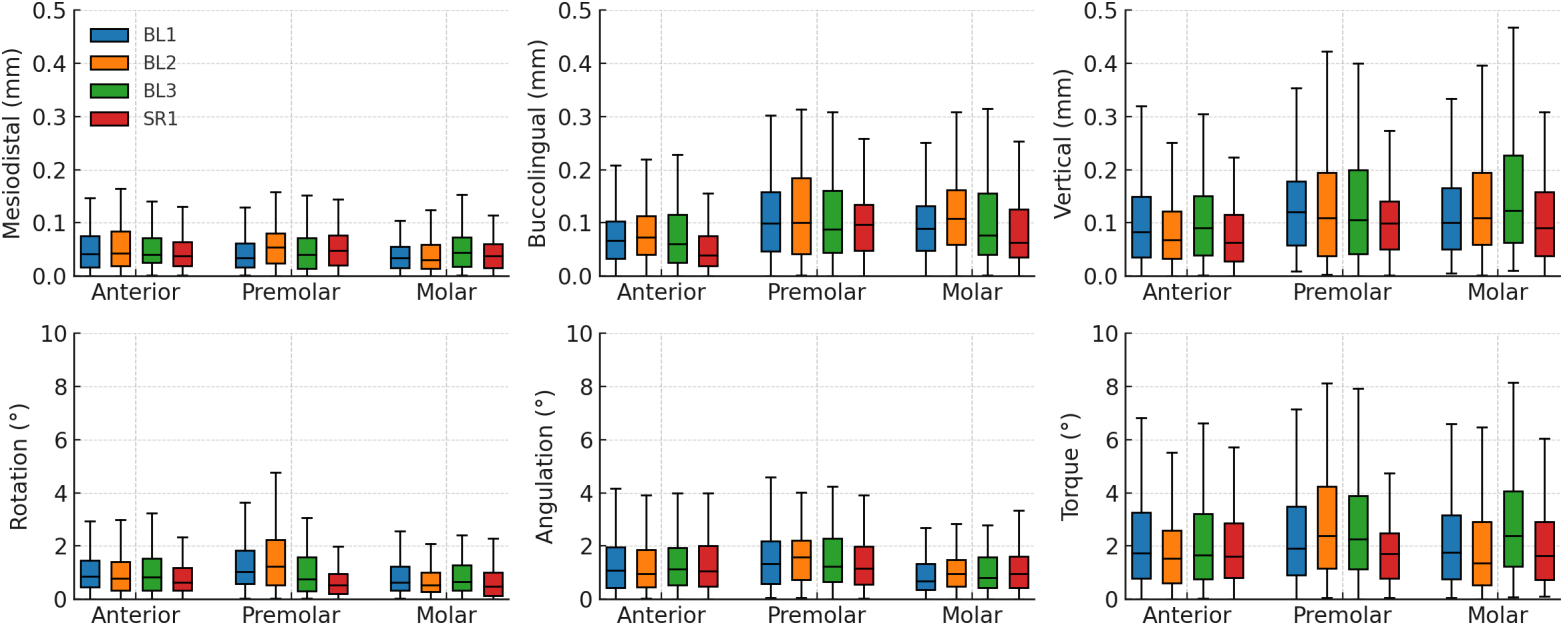
Box-and-whisker plots of absolute bracket transfer deviations by tooth group and tray design. BL1, bilayer tray (1.0-mm soft + 1.0-mm rigid); BL2, 2.0-mm soft + 1.0-mm rigid; BL3, 3.0-mm soft + 1.0-mm rigid; SR1 = single-layer 1.0-mm rigid tray.

Across all trays and tooth groups, absolute linear deviations were well below the 0.5-mm clinical threshold (one-sided Wilcoxon signed-rank tests, P < 0.05). Median mesiodistal deviations ranged from 0.03 to 0.05 mm, buccolingual deviations from 0.04 to 0.11 mm, and vertical deviations from 0.06 to 0.12 mm (Table 3 and Fig 4). Rotation and angulation were clinically acceptable across all trays, with median rotation ≤1.0° and median angulation around 1.0°–1.6°, both consistently below the 2.0° limit in all subgroups (P < 0.05). Torque remained the limiting dimension. Torque medians were typically between 1.5° and 2.4°, but with wide dispersion, and most subgroups did not demonstrate torque within the 2.0° limit (P > 0.05). Only a few subgroups achieved torque medians below the 2.0° limit with statistical significance, including BL2 anterior (1.53°, P = 0.007), SR1 premolars (1.71°, P = 0.036), and the SR1 total arch (1.65°, P = 0.012) (Table 4).

**Table 3:** Absolute bracket linear transfer deviations (mm) by tray design and tooth group.

Tray	Tooth group	Mesiodistal (mm)	Buccolingual (mm)	Vertical (mm)
BL1	Anterior (190)	0.04 (0.02–0.08) 0.05 ± 0.04	0.07 (0.03–0.10) 0.08 ± 0.07	0.08 (0.04–0.15) 0.10 ± 0.09
	Premolar (104)	0.03 (0.02–0.06) 0.04 ± 0.04	0.10 (0.05–0.16) 0.11 ± 0.08	0.12 (0.06–0.18) 0.13 ± 0.09
	Molar (131)	0.03 (0.02–0.06) 0.04 ± 0.04	0.09 (0.05–0.13) 0.10 ± 0.07	0.10 (0.05–0.17) 0.12 ± 0.10
	Total (425)	0.04 (0.02–0.06) 0.05 ± 0.04	0.07 (0.04–0.13) 0.09 ± 0.08	0.10 (0.04–0.16) 0.12 ± 0.09
BL2	Anterior (187)	0.04 (0.02–0.08) 0.06 ± 0.06	0.07 (0.04–0.11) 0.09 ± 0.08	0.07 (0.03–0.12) 0.09 ± 0.10
	Premolar (106)	0.05 (0.02–0.08) 0.06 ± 0.04	0.10 (0.04–0.18) 0.14 ± 0.12	0.11 (0.04–0.19) 0.13 ± 0.12
	Molar (134)	0.03 (0.01–0.06) 0.04 ± 0.04	0.11 (0.06–0.16) 0.12 ± 0.09	0.11 (0.06–0.19) 0.13 ± 0.10
	Total (427)	0.04 (0.02–0.08) 0.05 ± 0.05	0.09 (0.04–0.15) 0.11 ± 0.09	0.09 (0.04–0.17) 0.12 ± 0.11
BL3	Anterior (184)	0.04 (0.03–0.07) 0.06 ± 0.05	0.06 (0.02–0.12) 0.07 ± 0.06	0.09 (0.04–0.15) 0.11 ± 0.10
	Premolar (100)	0.04 (0.01–0.07) 0.05 ± 0.04	0.09 (0.04–0.16) 0.11 ± 0.08	0.11 (0.04–0.20) 0.13 ± 0.11
	Molar (134)	0.04 (0.02–0.07) 0.06 ± 0.06	0.08 (0.04–0.16) 0.11 ± 0.09	0.12 (0.06–0.23) 0.16 ± 0.14
	Total (418)	0.04 (0.02–0.07) 0.05 ± 0.05	0.07 (0.03–0.14) 0.09 ± 0.08	0.10 (0.04–0.19) 0.13 ± 0.12
SR1	Anterior (186)	0.04 (0.02–0.06) 0.05 ± 0.04	0.04 (0.02–0.08) 0.06 ± 0.06	0.06 (0.03–0.12) 0.09 ± 0.09
	Premolar (93)	0.05 (0.02–0.08) 0.06 ± 0.05	0.10 (0.05–0.13) 0.10 ± 0.07	0.10 (0.05–0.14) 0.12 ± 0.12
	Molar (133)	0.04 (0.02–0.06) 0.04 ± 0.04	0.06 (0.04–0.12) 0.08 ± 0.07	0.09 (0.04–0.16) 0.11 ± 0.10
	Total (412)	0.04 (0.02–0.07) 0.05 ± 0.04	0.06 (0.03–0.11) 0.08 ± 0.07	0.08 (0.03–0.14) 0.10 ± 0.10

Each cell shows the median (IQR) and the mean ± SD. NS, bracket deviation not within the 0.5-mm limit as determined by one-sided Wilcoxon signed-rank tests. BL1, bilayer tray (1.0-mm soft + 1.0-mm rigid); BL2, 2.0-mm soft + 1.0-mm rigid; BL3, 3.0-mm soft + 1.0-mm rigid; SR1 = single-layer 1.0-mm rigid tray.

**Table 4:** Absolute bracket angular transfer deviations (°) by tray design and tooth group.

Tray	Tooth group	Rotation (°)	Angulation (°)	Torque (°)
BL1	Anterior (190)	0.87 (0.45–1.46) 1.09 ± 0.92	1.09 (0.44–1.96) 1.43 ± 1.28	1.73 (0.77–3.26)^NS^ 2.22 ± 1.89
	Premolar (104)	1.04 (0.58–1.84) 1.28 ± 1.01	1.33 (0.58–2.18) 1.76 ± 1.67	1.90 (0.92–3.49)^NS^ 2.66 ± 2.61
	Molar (131)	0.64 (0.34–1.23) 0.92 ± 0.84	0.68 (0.36–1.34) 0.93 ± 0.78	1.76 (0.77–3.16)^NS^ 2.29 ± 2.02
	Total (425)	0.84 (0.42–1.50) 1.08 ± 0.92	1.02 (0.42–1.79) 1.36 ± 1.30	1.76 (0.79–3.24)^NS^ 2.35 ± 2.13
BL2	Anterior (187)	0.78 (0.33–1.41) 1.01 ± 0.93	0.95 (0.45–1.87) 1.45 ± 1.56	1.53 (0.60–2.59) 1.94 ± 1.74
	Premolar (106)	1.23 (0.52–2.23) 1.56 ± 1.37	1.58 (0.73–2.21) 1.76 ± 1.53	2.38 (1.16–4.25)^NS^ 3.14 ± 2.99
	Molar (134)	0.53 (0.27–1.01) 0.83 ± 0.92	0.95 (0.48–1.48) 1.19 ± 1.11	1.37 (0.53–2.91)^NS^ 2.13 ± 2.21
	Total (427)	0.74 (0.33–1.46) 1.09 ± 1.09	1.05 (0.52–1.89) 1.45 ± 1.44	1.66 (0.71–3.16)^NS^ 2.30 ± 2.30
BL3	Anterior (184)	0.84 (0.33–1.54) 1.09 ± 1.04	1.14 (0.53–1.93) 1.51 ± 1.47	1.67 (0.76–3.21)^NS^ 2.22 ± 2.04
	Premolar (100)	0.75 (0.30–1.59) 1.09 ± 1.03	1.24 (0.65–2.27) 1.61 ± 1.30	2.27 (1.14–3.89)^NS^ 2.79 ± 2.21
	Molar (134)	0.67 (0.32–1.29) 0.93 ± 0.98	0.81 (0.44–1.59) 1.19 ± 1.31	2.39 (1.24–4.07)^NS^ 3.08 ± 2.48
	Total (418)	0.70 (0.31–1.48) 1.04 ± 1.02	1.02 (0.52–1.90) 1.43 ± 1.38	2.06 (0.98–3.68)^NS^ 2.63 ± 2.26
SR1	Anterior (186)	0.63 (0.33–1.19) 0.87 ± 0.78	1.07 (0.48–2.02) 1.42 ± 1.27	1.60 (0.81–2.87)^NS^ 2.11 ± 1.88
	Premolar (93)	0.54 (0.22–0.95) 0.69 ± 0.62	1.17 (0.55–2.00) 1.40 ± 1.09	1.71 (0.79–2.48) 2.09 ± 2.15
	Molar (133)	0.47 (0.13–1.01) 0.69 ± 0.69	0.95 (0.44–1.61) 1.17 ± 0.92	1.65 (0.74–2.92)^NS^ 2.16 ± 1.94
	Total (412)	0.58 (0.25–1.07) 0.77 ± 0.72	1.06 (0.49–1.84) 1.33 ± 1.13	1.65 (0.78–2.81) 2.12 ± 1.96

Each cell shows the median (IQR) and the mean ± SD. NS, bracket deviation not within the 2.0° limit as determined by one-sided Wilcoxon signed-rank tests. BL1, bilayer tray (1.0-mm soft + 1.0-mm rigid); BL2, 2.0-mm soft + 1.0-mm rigid; BL3, 3.0-mm soft + 1.0-mm rigid; SR1 = single-layer 1.0-mm rigid tray.

**Fig 5:**
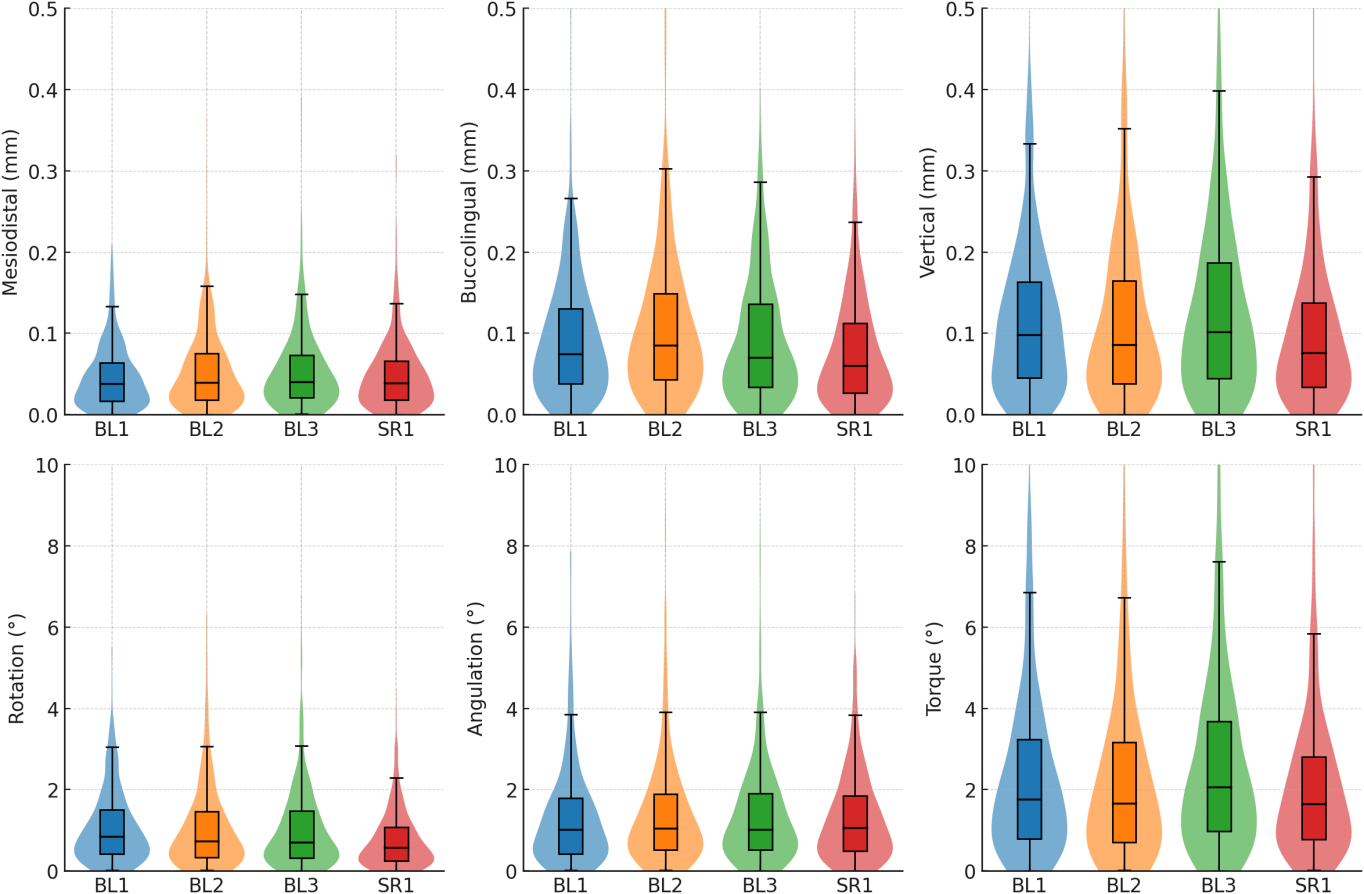
Violin and box plots of absolute bracket transfer deviations pooled across the total sample for each tray design. BL1, bilayer tray (1.0-mm soft + 1.0-mm rigid); BL2, 2.0-mm soft + 1.0-mm rigid; BL3, 3.0-mm soft + 1.0-mm rigid; SR1 = single-layer 1.0-mm rigid tray.

Between-tray differences are summarized in Table 5 and Figure 5. Across tooth groups, SR1 consistently ranked best for buccolingual (anterior and total P < 0.001) and rotation (premolar and total P < 0.001), while BL2 tended to rank worst for buccolingual (molar P = 0.01, total P < 0.001). Torque showed the largest tray effect in posterior segments: BL3 ranked worst in molars (P < 0.001) and overall (P = 0.005), while premolars showed higher torque errors with BL2 and BL3 compared to BL1 and SR1 (P = 0.027). Mesiodistal differences were limited to premolars (P = 0.040), where BL2 showed greater deviation than the other trays. For vertical positioning, tray effects were minor in the anterior segment (P = 0.017), but became pronounced in molars (P = 0.008) and overall (P < 0.001), where BL3 consistently performed worse than other groups. Angulation showed no meaningful tray effect (all P ≥ 0.143).

**Table 5:** Between-tray comparisons of absolute bracket-transfer deviations by tooth group and total arch.

Variable	Anterior	Premolar	Molar	Total
Mesiodistal	P = 0.474	P = 0.040 BL1^a^, BL2^b^, BL3^a^, SR1^a^	P = 0.074	P = 0.141
Buccolingual	P < 0.001 BL1^b^, BL2^b^, BL3^b^, SR1^a^	P = 0.489	P = 0.010 BL1^a^, BL2^b^, BL3^a^, SR1^a^	P < 0.001 BL1^b^, BL2^c^, BL3^b^, SR1^a^
Vertical	P = 0.017 BL1^a^, BL2^a^, BL3^a^, SR1^a^	P = 0.645	P = 0.008 BL1^a^, BL2^a^, BL3^b^, SR1^a^	P < 0.001 BL1^b^, BL2^a^, BL3^b^, SR1^a^
Rotation	P = 0.103	P < 0.001 BL1^b^, BL2^c^, BL3^b^, SR1^a^	P = 0.037 BL1^a^, BL2^a^, BL3^a^, SR1^a^	P < 0.001 BL1^b^, BL2^b^, BL3^b^, SR1^a^
Angulation	P = 0.744	P = 0.387	P = 0.143	P = 0.673
Torque	P = 0.514	P = 0.027 BL1^a^, BL2^b^, BL3^b^, SR1^a^	P < 0.001 BL1^a^, BL2^a^, BL3^b^, SR1^a^	P = 0.005 BL1^a^, BL2^a^, BL3^b^, SR1^a^

Each cell reports the Kruskal–Wallis P; when P < 0.05, trays are ranked with superscripts from adjusted pairwise Mann–Whitney tests (Benjamini–Hochberg). Different letters indicate significant pairwise differences. BL1, bilayer tray (1.0-mm soft + 1.0-mm rigid); BL2, 2.0-mm soft + 1.0-mm rigid; BL3, 3.0-mm soft + 1.0-mm rigid; SR1 = single-layer 1.0-mm rigid tray.

## Discussion

This prospective cohort study evaluated whether design features of thermoformed IDB trays for lingual appliances meaningfully affect bracket transfer accuracy at the tooth level. We tested two null hypotheses: first, that neither translational nor rotational deviations would differ among the four tray designs; and second, that any residual deviation would remain within clinically acceptable thresholds. The primary findings only partially support that hypothesis, with statistically relevant design effects emerging for buccolingual, vertical, rotation, and torque. On the one hand, all trays delivered within-limit linear accuracy and clinically acceptable control for rotation and angulation. Torque remained the critical limiting variable overall: although some subgroups achieved median torque error below the 2.0° limit, most tray–tooth combinations did not. Taken together, these results reject a strict no-difference model and instead indicate that thermoformed architecture influences how precisely brackets are seated in clinically sensitive planes, even though all four tray types broadly satisfied conventional acceptability thresholds for lingual bonding.

The observed superiority of the single-layer rigid tray in several key dimensions can be interpreted in terms of mechanical stability and seating control during transfer. Because SR1 liner is stiffer than the bilayer designs, it is less susceptible to elastic deformation when finger pressure is applied intraorally, allowing the tray to preserve its original geometry and clamp the bracket in its intended position rather than distorting around it. The rigidity also produces a more secure “grip” on the bracket body, which likely contributes to the lower rotational deviations. In addition, SR1 was the thinnest tray, which improves direct visual control at bonding: the operator can more easily verify that the tray is fully seated against the lingual surfaces, reducing the risk of an incompletely seated posterior segment [7]. These same arguments also help explain the performance gradient observed among the bilayer trays. BL1 tended to outperform BL2 and BL3, consistent with the idea that adding a thicker inner liner increases overall bulk and flexibility, both of which can permit slight deformation and micro-shifting under load. BL1 also enclosed the bracket more completely, including the undercuts beneath the tie wings, which likely stabilized the bracket more effectively than the less retentive geometries of BL2 and BL3. By contrast, differences among tray types were minimal in the mesiodistal direction and for angulation. This is probably because mesiodistal translation and crown angulation are primarily constrained by the rigid vertical walls of the tray pocket, so these dimensions depend less on bulk, flexibility, or seating pressure.

When placed in the context of existing reports on indirect lingual bonding, the present results indicate a meaningful improvement in several dimensions of transfer accuracy. Schubert et al. reported linear deviations of 0.10–0.13 mm and angular deviations of 2.20–3.21° using the Quick Modul System, with all angular measurements exceeding the 2.0° limit [17]. One likely reason for this comparatively poorer accuracy is the use of individually bonded brackets in that system, which provides fewer intra-arch reference constraints than a segmental transfer approach. A separate study using double thermoformed trays found that, although rotational control was within the 2.0° limit, crown angulation still remained outside the range [6]. At the same time, the observed pattern–good overall accuracy but torque frequently emerging as the weakest axis and often exceeding 2.0°–is broadly consistent with more recent reports on digitally guided lingual bonding [7,11,18]. Interestingly, our finding that the fully encapsulated bilayer tray (BL1) outperformed designs that exposed the gingival tie wing (BL2 and BL3) contrasts with a previous investigation of 3D-printed rigid trays, in which an open-pocket design was more accurate than a fully enclosed one [11]. This apparent discrepancy can be explained by material behavior: in rigid printed trays, full encapsulation can act as a hard lid that prevents the bracket base from fully seating in the pocket, whereas in the present study, the inner liner was flexible. In other words, when the lining material is deformable rather than rigid, full encapsulation becomes a retentive aid instead of a seating obstacle.

When our lingual results are benchmarked against recent IDB data for labial brackets, two patterns emerge. First, absolute linear accuracy is now essentially comparable between lingual and labial IDB workflows [19]. Sabbagh et al. reported mean linear deviations in the 0.04–0.21 mm range, with posterior teeth showing slightly larger mesiodistal discrepancy (0.18–0.20 mm), which was attributed to the difficulty of maintaining uniform seating pressure in the distal segments [20]. Consistent with this, Bachour et al. reported mean mesiodistal and vertical differences of 0.26 mm and 0.28 mm, respectively [21]. Second, angular transfer remains the main challenge, especially torque [22]. Recent studies report angular errors in the range of 0.20–1.75° overall, but torque frequently exceeded the 2.0° clinical limit, particularly with less retentive designs and inexperienced operators [20]. In addition, Zhu et al. confirmed that vertical positioning remains the weakest linear axis, with vertical discrepancies of ≈0.28 mm being the largest among all linear directions, despite mesiodistal and buccolingual control remaining below 0.20 mm [23]. Finally, a split-mouth randomized clinical trial in vivo showed that fully digital labial IDB reduced translational error by 0.34 mm and reduced orientation error by 4.8° relative to conventional direct bonding, while also highlighting that angular deviations under IDB can remain above the 2.0° limit [24]. Taken together, the present lingual data align with the linear ranges now reported for labial IDB, but they reproduce the same global limitation seen in the labial literature: torque and posterior vertical seating remain the hardest dimensions to transfer accurately [25].

From a clinical standpoint, these findings suggest that tray selection can be guided by a balance of cost, chairside efficiency, and dimensional fidelity rather than by assuming that thicker trays are more precise. All four tray designs in this study delivered bracket positions that were largely within clinical thresholds, meaning any of them are viable for routine lingual bonding. However, SR1 showed the most favorable buccolingual and rotational control and frequently produced the lowest torque error, but at the cost of longer bonding time per arch. Delivery time did not appear to be driven primarily by undercut engagement or blockout; instead, the pattern observed suggests that material stiffness and tray thickness may play a larger role in retention and disengagement across designs. Because SR1 uses a single rigid material with minimal thickness, it is also the least expensive to fabricate. BL1 represents a practical compromise: although its accuracy was slightly inferior to SR1 in some axes, it remained clinically acceptable across most dimensions and could be seated and removed more quickly, which is attractive in situations where chairtime is critical. By contrast, increasing liner thickness in BL2 and BL3 did not translate into measurable improvements in precision; in fact, thicker bilayer designs tended to worsen torque and vertical control in posterior segments while adding material cost and tray bulk. These data argue against routinely escalating to thicker bilayer constructions on the assumption that “more support” improves transfer. If the clinical priority is maximum positional fidelity, such as in cases requiring strict control of rotation and buccolingual position, SR1 is justified despite the added delivery time, whereas if the priority is procedural speed with acceptable accuracy in straightforward alignment cases, BL1 remains a reasonable option.

This study has several limitations that should be considered when interpreting the findings. First, although all trays were tested clinically under routine full-arch lingual bonding, residual in vivo heterogeneity remained. Baseline crowding patterns differed between patients and may have altered tray seating despite no significant difference in crowding severity among groups [5,12]. Second, the protocol evaluated segmental delivery rather than the clinically common scenario of rebonding a single failed bracket, which is typically more challenging because the tray no longer benefits from multi-tooth indexing. Therefore, the present data cannot be directly extrapolated to isolated rebonding. Third, all bonding procedures were performed by a single calibrated operator using one digital workflow, one scanner, and one set of tray fabrication parameters. Operator experience, scanner accuracy, and material processing can vary between clinics and could shift the relative performance of the tray designs. Finally, accuracy was assessed immediately after transfer in terms of nominal linear and angular deviations from the virtual setup. The study did not evaluate how these deviations propagate clinically, such as whether an initial 2–3° torque loss at bonding is later compensated by bracket slot–archwire play [26–28]. As a result, the biological relevance of small residual errors remains inferential rather than outcome-based. Future studies should incorporate multi-operator, multi-center bonding (including single-tooth bonding), and longitudinal follow-up to determine which transfer errors actually persist to finishing and require correction.

### Conclusions

In summary, all four thermoformed transfer trays produced acceptable IDB accuracy for lingual brackets under routine chairside conditions, with all linear deviations, rotational, and angulation errors generally within the clinical limits. However, tray design influenced specific error patterns: the single-layer rigid tray (SR1) showed the most favorable buccolingual and rotation control but required longer chairside delivery, whereas the thinner bilayer tray (BL1) with full bracket encapsulation provided acceptable accuracy with shorter delivery time. Increasing liner thickness (BL2, BL3) did not improve precision and was associated with greater deviation in some dimensions. Across all designs, torque remained the most error-prone dimension. Clinically, SR1 may be preferred in cases requiring strict control of rotation and buccolingual position, whereas BL1 offers a faster alternative for straightforward alignment without routine escalation to thicker bilayer trays.

## Supporting information

S1 File Dataset. (XLSX)
